# Twelve-Month Evaluation of Temperature Effects of Radiotherapy in Patients after Mastectomy

**DOI:** 10.3390/ijerph19052834

**Published:** 2022-02-28

**Authors:** Agnieszka Baic, Dominika Plaza, Barbara Lange, Łukasz Michalecki, Agata Stanek, Krzysztof Ślosarek, Armand Cholewka

**Affiliations:** 1 Faculty of Science and Technology, University of Silesia, 75 Pułku Piechoty Street 1A, 41-500 Chorzow, Poland; armand.cholewka@gmail.com; 2 Radiotherapy Planning Department, Maria Skłodowska—Curie National Research Institute of Oncology Gliwice Branch, Wybrzeze Armii Krajowej Street 15, 44-102 Gliwice, Poland; dominikaplaza1@gmail.com (D.P.); krzysztof.slosarek@io.gliwice.pl (K.Ś.); 3 IIIrd Radiotherapy and Chemotherapy Department, Maria Skłodowska-Curie National Research Institute of Oncology Gliwice Branch, Wybrzeze Armii Krajowej Street 15, 44-102 Gliwice, Poland; barbara.lange@io.gliwice.pl; 4 Department of Radiation Oncology, University Clinical Center, Medical University of Silesia in Katowice, Ceglana Street 35, 40-514 Katowice, Poland; lmichalecki@uck.katowice.pl; 5 Chair and Clinical Department of Internal Medicine, Angiology and Physical Medicine, Faculty of Medical Sciences in Zabrze, Medical University of Silesia, Batorego Street 15, 41-902 Bytom, Poland; astanek@tlen.pl

**Keywords:** radiation therapy, thermography, breast cancer

## Abstract

The aim of this study was to verify the changes in the temperature distribution within the breast at twelve months after the end of radiotherapy for breast cancer. The study included twenty-four women. The first test group consisted of twelve women who underwent breast mastectomy and qualified for radiotherapy according to standard medical treatment procedures. The second group included twelve healthy women. The tests were conducted before treatment with radiation therapy and two months, six months, nine months, and one year after the end of treatment. The mean temperature values changed depending on the time that had elapsed since the end of treatment. The highest temperature increase in all patients was observed six months after the end of radiotherapy. This research has confirmed that the assessment of temperature changes in the breast area after radiotherapy can evaluate the severity and lesions in the time course of the radiation reaction.

## 1. Introduction

Thermal imaging has been used in medicine, biomedical engineering, and physical therapy for a few decades [1,2]. It allows the assessment of the body surface temperature distribution, which is valuable and useful, because local temperature changes can inform the physician about metabolism changes and thus indirectly represent the pathological processes in the human body. Therefore, for the correct analysis of camera images, it is essential to know the physiology and anatomy of the human body. Infrared thermography is a scientific field that uses the properties of electromagnetic radiation in the infrared range to measure the temperature of the surface of objects. This analysis is based on the fact that everything and everyone has a temperature higher than absolute zero (0 K) [2]. Therefore, everything is emitting radiation, and this energy is related to the temperature of the object and the radiation wavelength. Accordingly, the human body also emits the radiation in the infrared range, which can be imaged by a thermal camera. The effect of this phenomenon is to create an image in the form of a thermogram, which is a visual indicator of the amount of infrared energy emitted or reflected by the tissue [3,4,5,6].

The examined tissue is compared to the so-called perfectly black body (absorption coefficient equal to 1.0), which absorbs 100% incident radiation, and the so-called perfectly white body, which does not emit radiation, instead reflecting it completely. The thermal imaging camera shows the exact value and distribution of temperature, which is connected to the vascularity and metabolism of the tissue. Through establishing measurement standards for thermography, developing computer technologies, and constructing sensitive thermovision detectors, thermograms have become increasingly frequent objects of interest as a diagnostic tool in various fields of medicine [1,2]. Currently, in medicine, a thermal imaging camera is often used to assess the extent and intensity of inflammation in examined tissue based on the phenomenon of high thermoemission, which means that the local blood supply or tissue metabolism increased [7,8]. The great advantage of thermovision is its noninvasiveness, which allows repeated harmless testing without the risk of any side effects. An additional factor contributing to the expansion of thermal imaging research is the affordability of thermal imaging cameras. The devices are widely available and easy to use. The increased usage of thermal imaging tests has necessitated the introduction of new standards concerning both the conduct of the tests themselves and the equipment requirements. The proper preparation of patients and the research room is extremely important. According to the guidelines of the European Association of Thermology, the room where the thermovision measurements take place should support the convenient placement of the measuring devices and visualization of the entire studied area [4,5,6]. An additional requirement is constant temperature and humidity. Before the test, each participant should acclimatize to the temperature of the room. This process should take several minutes. The participant should not touch their body during acclimatization and should remain at rest. The patient must prepare for the examination. On the day of measurement, he/she must not exercise or be under the influence of alcohol or drugs, and must be healthy (not have infections that lead to an increase in body temperature above 37 °C and not taking medications that could cause a change in body temperature) [7]. On the day of the examination, no cosmetics should be used on the skin and it should not be exposed to sunlight. Patients with dermatological changes on the skin, fresh tattoos, or scars were also not suitable for thermal imaging examinations. We performed the tests at the same distance, which should not be less than 1 m. For the analysis of thermographic examinations, a special program should be used, the operation of which should be taught by trained personnel. It is extremely important to take into account all factors that may affect the temperature distribution when performing thermal imaging studies. When all these conditions are met, the tests carried out are of the highest quality [1,2,3,4,5,6,7].

Despite the development of technology and the uptake of newer treatment methods, neoplastic diseases still rank second among the causes of death in the world [9]. Cardiovascular diseases are the only diseases with higher mortality than cancers. The detection of tumors at an early stage, as well as the use of appropriate treatment, sometimes allows for complete remission [9].

Breast cancer is a malignant tumor that originates in the cells of the epithelium that line the inside of the lobules and milk ducts. Any change in the structure of the breast gland, thickening or wrinkling of the skin, and sudden asymmetry of the breast should be discussed with a specialist. The symptoms of breast cancer in more advanced stages may include locations associated with the formation of metastases. Usually, the axillary lymph nodes are affected first, which manifests as enlargement and swelling. A massive infiltration of this group of nodes hinders the outflow of lymph from the adjacent upper limb, resulting in its swelling. Subsequent metastases may involve nodes located in the supraclavicular fossa. Statistics show a constantly increasing trend in the incidence of breast cancer. Over the last 30 years, the number of cases in Poland has doubled. Women aged 50–69 are at the greatest risk of developing the disease. However, breast cancer is also increasingly prevalent in younger patients, aged 20–49 [10,11,12,13,14,15]. Surgery, radiotherapy (RT), and chemotherapy are methods used in the treatment of breast cancer. In women who undergo breast mastectomy—in the case of the worsening prognosis factors—chest wall irradiation is used with or without armpit and collarbone radiation [16,17]. Radiotherapy is used as an adjuvant treatment due to the high rate of recurrence in treated breast cancers, of up to 20% after 10 years. Radiotherapy uses ionizing radiation to destroy cancer cells. For this purpose, the patient is given the highest possible dose of ionizing radiation to the changed neoplastic tissues, while minimizing the dose that will be administered to healthy cells. This is possible owing to the use of specialized equipment for treatment planning, performance, and control of therapy using ionizing radiation. The critical organs (OAR; organs at risk) are the structures that are located close to the irradiated volume (the target). In breast cancer radiotherapy, the OARs include the lungs and the heart and, not infrequently, the head of the humerus. Critical organs have strictly defined tolerance doses, which, if exceeded, might result in radiation complications. Radiotherapy technology is a rapidly advancing technology [16,17,18]. The new devices for the implementation of treatment, as well as the use of modern techniques, such as IMRT (intensity modulated radiation therapy), i.e., beam intensity modulation or VMAT (volumetric modulated arc therapy), known in the Polish nomenclature as multi-arc therapy, achieved a significantly reduction in the side effects of radiation therapy. In patients with breast cancer treated with ionizing radiation therapy, side effects often occur, including a skin response manifested in the form of radiation reaction, e.g., skin redness [19,20,21]. The reaction resulting from radiotherapy is caused by the death of cells most exposed to ionizing radiation. Redness occurs in the area directly subjected to radiotherapy. Negative effects caused by radiotherapy may persist for several weeks to even several years after the end of treatment. Adverse radiation reactions appear most often in neoplasms located in the area of the head and neck, perineum, and breast. With the help of thermovision, which is a noninvasive and painless method, we can observe thermal asymmetry between the breasts, contributing to the early detection of disturbing symptoms. The use of infrared imaging in the control of patients after radiotherapy is aimed at assessing the effects of treatment and observing the negative effects of ionizing radiation, which affect patient comfort [22,23,24,25,26,27,28,29].

For this reason, the aim of this study was to check and compare the changes in temperature distribution in patients after mastectomy at various times after the end of radiotherapy.

## 2. Materials and Methods

The study design was approved by the Bioethics Committee of the Oncology Center-Maria Skłodowska-Curie Institute in Warsaw on 6 October 2016, which was confirmed in opinion No. 38/2016.

The first group included twelve patients who were qualified for radiotherapy. Twelve healthy participants enrolled in the study as members of the second group were people without reported health problems who had no history of breast cancer. Their participation in the study was voluntary. For the first group of patients, the mean age was 56 ± 9.8 years, body weight was 72 ± 11 kg, and height was 1.67 ± 0.07. In healthy participants, the mean age was 53 ± 9.7 years, body weight was 71 ± 12 kg, and height was 1.65 ± 0.05. Study patients who completed radiotherapy received a total dose of 50 Gy in 25 fractions. Due to the stage of the tumor, the patients were qualified for subcutaneous mastectomy with the removal of metastatic lymph nodes. Before the test, each participant was informed about its course and how to prepare for the test. Each participant had to consent to participate and fill in a participation questionnaire. In the questionnaire, the patient answered questions about their health condition. The overall intention was to perform several tests for which the participants had to give their written consent. Thermographic examinations were performed before the treatment with radiation therapy, and then after various periods from two months to one year after the end of treatment. The study used a FLIR System E60 thermal imaging camera with a detector resolution of 320 × 240 pixels and a thermal sensitivity of 0.05 K. Thermal imaging was performed in a specially prepared room; the temperature was constant at 22 ± 1 °C and the humidity ranged from 40% to 45% [2,3,4,5,6,7,8]. The relative air humidity in the measuring environment was low enough so that there was no condensation in the air (fog), on the measuring object, on the protective cap, or on the thermal imaging camera lens. If the lens (or the protective cap) becomes fogged, then some of the infrared radiation will not be picked up as it will not be able to fully penetrate the lens through the water. The same experimental design was performed on each patient. The time that was needed to prepare for the study lasted about twenty minutes. During this time, the patients did not wear upper garments. The examination was performed in a standing position with raised arms according to standard protocols [30,31,32,33,34,35,36,37,38].

The research was performed using the ThermaCAM Researcher Pro 2.10. The statistical analysis was performed using STATISTICA (with a confidence interval of 0.95) to interpret the results. To determine the statistical significance, Student’s *t*-test was performed. In addition, the temperature changes in the breast were analyzed after the end of radiotherapy by using Pearson coefficient. Thermal differences within the chest were analyzed for both healthy and treated women. For the group of women who underwent mastectomy, the changes in temperature distribution over time were analyzed. As several tests were performed, we were able to analyze the relationship between temperature changes and the time that had elapsed since the end of the treatment.

**Figure 1:**
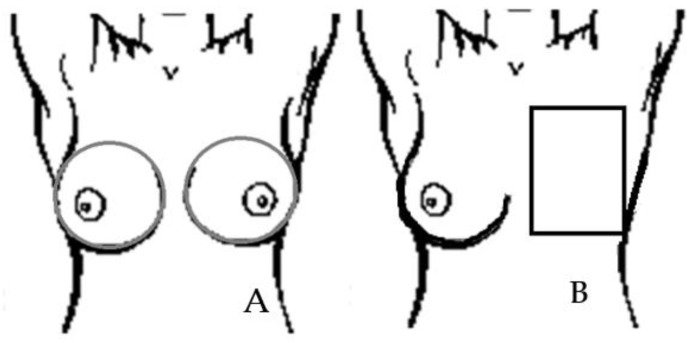
Schematic drawing of the breast area on all thermograms. (**A**) Healthy patients; (**B**) patients after mastectomy.

The area after mastectomy that was treated and analyzed is marked with a rectangle (Figure 1). Depending on the size and structure of the breasts, the rectangle was adapted to the anatomy of each patient. Below is an example diagram defining the area subject to further analysis.

**Figure 2:**
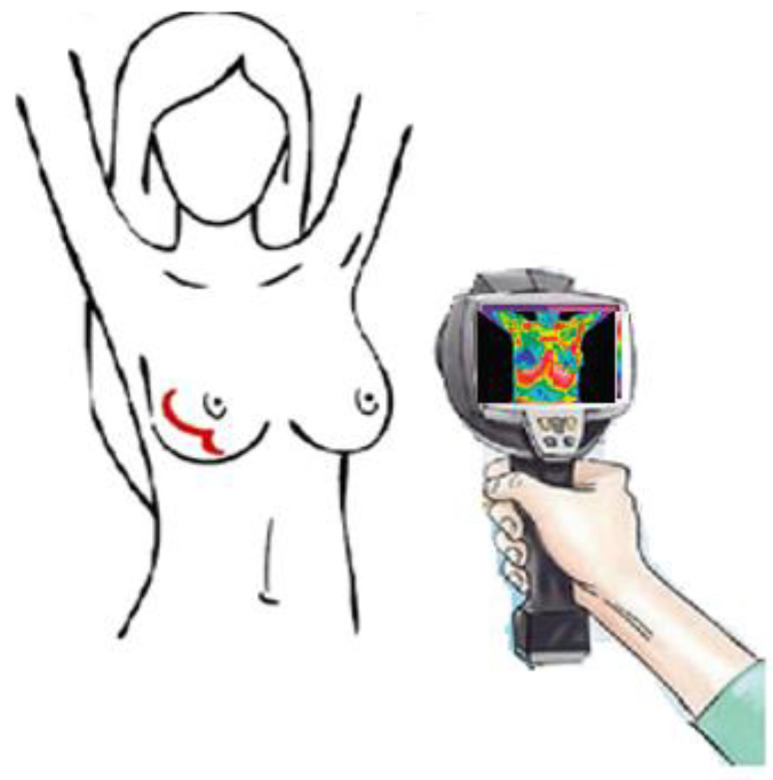
Simple scheme demonstrating the workflow and methodology of the study.

Figure 2 presents the workflow and methodology of the study.

## 3. Results and Discussion

**Figure 3:**
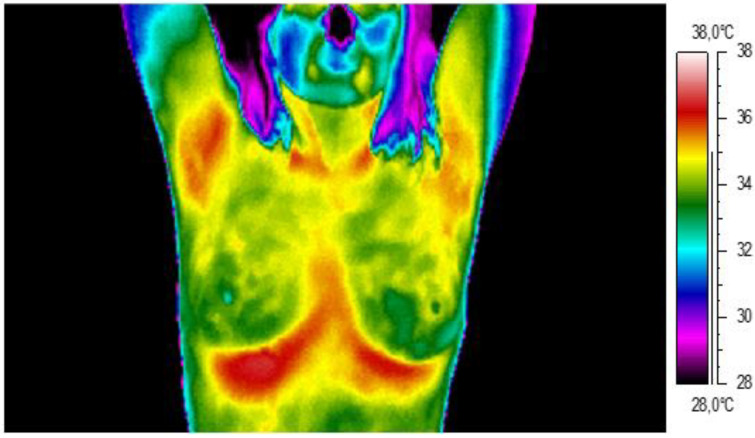
Thermogram of an example healthy participant.

In the group of healthy participants, the mean temperature was 32.61 °C for the left breast and 32.62 °C for the right breast. The average temperature difference between the analyzed areas was 0.23 °C. An example of a thermal image for a healthy patient is shown below (Figure 3). There is no thermal asymmetry in the breast area; the temperature of the nipple is close to that of the breast gland. The temperature range used for the analysis was between 28 °C and 38 °C.

**Figure 4:**
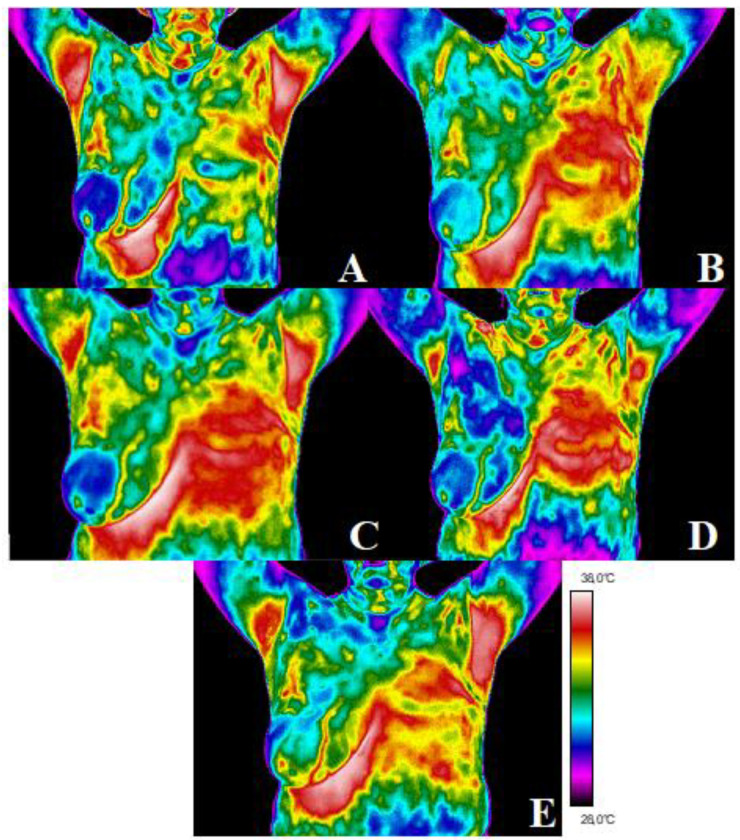
After mastectomy: before radiotherapy (thermogram (**A**)), two months after the end of radiotherapy (thermogram (**B**)), six months after the end of radiotherapy (thermogram (**C**)), nine months after the end of radiotherapy (thermogram (**D**)), and twelve months after the end of radiotherapy (thermogram (**E**)).

For the twelve patients in the test group who underwent mastectomy, five analyses were conducted: before radiotherapy (thermogram A), two months after the end of radiotherapy (thermogram B), six months after the end of radiotherapy (thermogram C), nine months after the end of radiotherapy (thermogram D), and twelve months after the end of radiotherapy (thermogram E). We can see in Figure 4 that there is strong thermal asymmetry between the healthy breast and the area that was subjected to radiotherapy. Two months after stopping the treatment, the area with elevated temperature is larger. Moreover, it reaches its highest value at six months after the end of treatment. At this time, there are also very frequent periods of repairing healthy tissues after they have received a dose of radiation. In the months following the end of treatment, the thermal asymmetry was still visible, but became smaller with each subsequent examination.

The mean temperatures for the analyzed area for all patients are presented in Table 1.

When analyzing the mean temperature values read from the thermograms, we can see that the temperature after radiotherapy is higher than before the treatment. In addition, there is a noticeable tendency for the highest value to be reached 6 months after the end of treatment, followed by a decrease in the average temperature. However, even after a year, the temperature measured is higher than before the treatment. The temperature differences are presented in Table 2. It can be observed that the highest difference in the mean value, in comparison to pretreatment measurement, occurs 6 months after radiotherapy and is 1.56 degrees Celsius.

**Figure 5:**
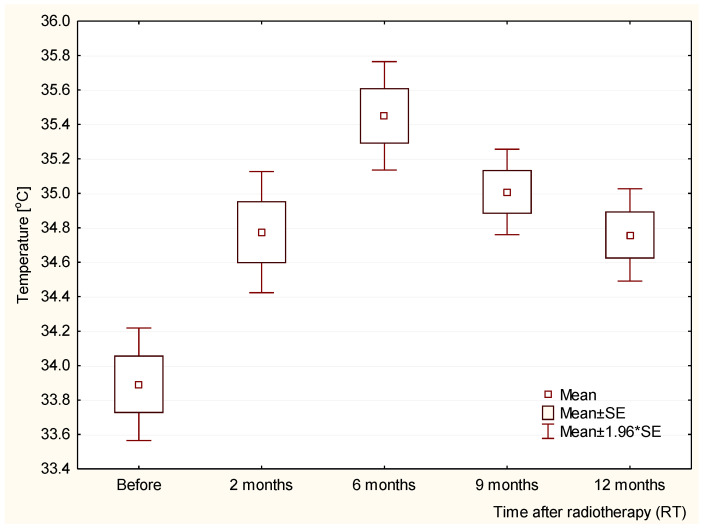
Temperature values for the group of patients: before RT, 2 months after RT, 6 months after RT, 9 months after RT, and 12 months after RT.

**Figure 6:**
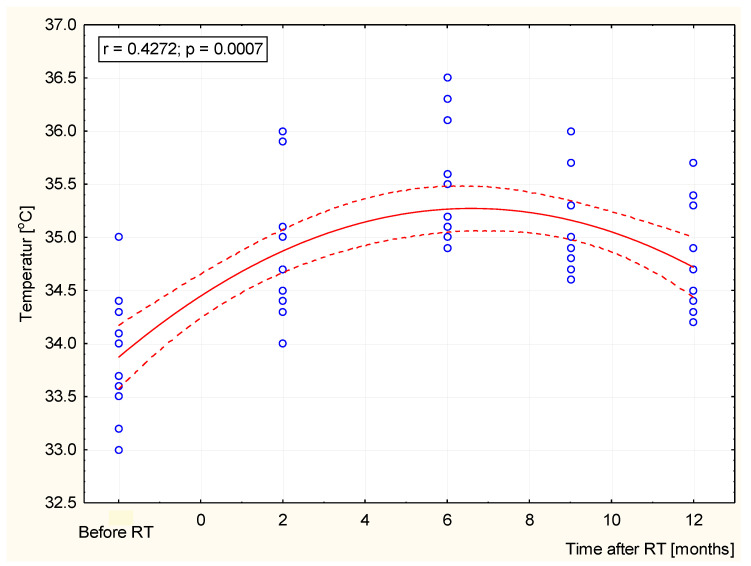
Temperature correlation between before radiotherapy and after radiotherapy (2, 6, 9, and 12 months).

In order to get a more complete picture and better insight into the obtained results, the collected data were statistically analyzed. The acquired results are presented in Figure 5 and Figure 6. The obtained values were statistically significant (*p* < 0.05) when comparing the values before RT with a specified number of months after radiotherapy (2, 6, 9, or 12 months). Statistical significance was obtained by comparing the values at 6 months after RT with individual months (2, 9, and 12 months).

The study showed a strong positive correlation up to the sixth month after RT, and a negative correlation (Figure 6) for values over 6 months after radiotherapy. The statistical analysis showed that the increase in the average temperature up to the sixth month is significant at every stage of the research, and the obtained values for the increase, up to 1.6 °C, were very high and certainly significant enough for thermovision diagnostics.

## 4. Discussion

Breast radiotherapy is a local treatment method that uses the energy of ionizing radiation. Unfortunately, it is not harmless to the patient. By developing modern techniques and newer equipment it is possible to reduce the dose received by healthy organs while irradiating the tumor at the same time. The side effects of radiotherapy are mainly fatigue, drowsiness, and skin reactions [39,40,41,42,43]. The skin reactions to the irradiation can be classified according to the common NCI CTCAE scale [44]. The values range from I to V and classifies patients depending on the intensity of the burn, from mild reddening (rated I) to death (rated V). Patients who participated in the study after radiotherapy were classified, according to the CTCAE scale, as grade I and II, which means that they developed a slight erythema or erythema of moderate intensity. The reactions usually appear before the end of treatment and disappear few months after the end of treatment. Its intensity and appearance depend on the patient’s age, the size of the irradiated area, the fractional dose, the total dose, and comorbidities. In addition to erythema, skin dryness and hair loss can occur. Discoloration may appear as a result of damage to skin cells. Peeling of the skin (dry then wet) may appear a few weeks after stopping treatment. These reactions include pigment disorders, skin fibrosis, vascular changes, and necrosis; thus, changes in the body and skin temperature indicate that thermal imaging is an objective and completely safe imaging method for patient evaluation.

This paper aimed to present the use of thermal imaging to describe the thermal response of breast tissue to radiation therapy. This may be a new method that is useful to control the risk of developing radiation dermatitis. Previous studies have shown that the incidence of skin toxicity is rising after radiotherapy, but do not present the changes that appeared in individual months after radiotherapy or time how long they persist on the skin [22,24,30,32,33,34].

It has been shown that thermography allows the quantification of temperature fluctuations in the irradiated area in the example of adjuvant radiotherapy in patients after mastectomy for breast cancer, so it can be used in clinical practice. The mean temperature of the breast area obtained two months after the end of treatment was nearly 0.9 °C higher than before treatment. After another control, which took place six months after the end of the treatment, it was noticed in all the patients that the mean temperature increased again to a value 1.56 °C higher than the temperature before radiotherapy, which may be the result of persistent local inflammation in the course of the radiation reaction. After this time, the temperature dropped in subsequent tests, but even after a year it did not reach the same value as before the treatment. For the study groups, the correlations between temperature values and time after the end of radiotherapy were determined. Figure 4 presents the relationship between the parameters, which showed that the temperature values increased with the length of time after radiotherapy. The Pearson correlation coefficient was 0.43; moreover, the relationships were statistically significant, with *p* < 0.05. The trend of the reduced the temperature at 6 months after RT was observed, which may relate to the healing process. The general recovery of irradiated tissue will probably lead to temperature equalization over the long-term follow-up period [45]. However, late radiation effects may change the local microenvironment and tissue metabolism owing to persistent subtle differences in temperature. Thermal imaging seems to be helpful in quantitative monitoring of dermatitis related to radiotherapy. Due to the applied that methodology, it is possible to monitor the local persistence of inflammation. This assumes the importance of considering that a chronic inflammatory state in soft tissues, for example the adipose tissue, could have adverse effects, such as oncogenesis itself [46]. We can observe that tissue regeneration processes are long lasting and change over time [47,48,49,50].

## 5. Conclusions

The conducted study confirmed that:

1. Thermography allows the assessment of the dynamics of temperature changes in the tissue after radiotherapy in the mastectomy area;
2. The observed temperature fluctuations are the most probable effects of radiation changes in the tissues in the course of the radiation reaction and the healing process;
3. The quantitative assessment of temperature fluctuations in the irradiated area, as one of the parameters of the intensification of the radiation reaction, may be used in clinical practice and in research studies that assess the effectiveness of pharmaceuticals used in the treatment of radiation symptoms;
4. The small number of observations has limited the conclusions. The expansion of the research into the use of thermography in clinical practice is needed.

## Figures and Tables

**Table 1:** Results obtained for the test group in the study.

	Before RT [°C]	2 Months after RT [°C]	6 Months after RT [°C]	9 Months after RT [°C]	12 Months after RT [°C]
**Patient 1**	34.0	34.5	36.3	34.7	34.5
**Patient 2**	34.4	35.1	35.5	34.8	34.3
**Patient 3**	34.1	34.7	35.0	34.6	34.2
**Patient 4**	35.0	36.0	36.5	36.0	35.7
**Patient 5**	34.4	35.9	36.1	35.7	35.4
**Patient 6**	34.3	35.0	35.6	35.3	35.3
**Patient 7**	33.5	34.4	35.0	34.9	34.7
**Patient 8**	33.0	34.5	35.1	34.7	34.5
**Patient 9**	33.5	34.3	34.9	34.7	34.4
**Patient 10**	33.7	34.4	35.2	35.0	34.9
**Patient 11**	33.6	34.5	35.1	34.8	34.5
**Patient 12**	33.2	34.0	35.1	34.9	34.7

**Table 2:** Temperature difference at different times from the end of radiotherapy compared with the pretreatment study.

2 Months after RT [°C]	6 Months after RT [°C]	9 Months after RT [°C]	12 Months after RT [°C]
**0.88**	1.56	1.12	0.87

## Data Availability

Not applicable.

## References

[1] Podbielska H., Skrzek A. (2014). Biomedyczne Zastosowania Termowizji.

[2] Ammer K., Ring E.F.J. (1995). The Thermal Image in Medicine and Biology.

[3] Ring E.F.J. (1998). Progress in the measurement of human body temperature. IEEE Eng. Med. Biol. Mag..

[4] Ring E.F.J., Ammer K. (2000). The Technique of Infrared Imaging in Medicine. Thermol. Int..

[5] Ring E.F.J., Ammer K. (2012). Infrared thermal imaging in medicine. Physiol. Meas..

[6] Ammer K. (2008). The Glamorgan Protocol for recording and evaluation of thermal images of the human body. Thermol. Int..

[7] Bauer J., Hurnik P., Zdziarski J., Mielczarek W., Podbielska H. (1997). Thermovision and its applications in medicine. Acta Bio. Opt. Inf. Med..

[8] Chmielewski L., Kulikowski L.J., Nowakowski A. (2003). Obrazowanie Biomedyczne.

[9] Bojakowska U., Kalinowski P., Kowalska M.E. (2016). Epidemiologia i profilaktyka raka piersi = Epidemiology and prophylaxis of breast cancer. J. Educ. Health Sport..

[10] Kopans D.B. (1984). “Early” breast cancer detection using techniques other than mammography. Am. J. Roentgenol..

[11] Ng E.Y.K. (2009). A review of thermography as promising non-invasive detection modality for breast tumor. Int. J..

[12] Śniadecki M. (2015). Kryteria Rzpoznawania I Wczesne Objawy Chorób Nowotworowych.

[13] Ellis L.M., Fidler I.J. (1995). Angiogenesis and breast cancer metastasis. Lancet.

[14] Fox S.B., Generali D.G. (2007). Breast tumour angiogenesis. Breast Cancer Res..

[15] Gamagami P. (1986). Atlas of Mammography: New Early Signs in Breast Cancer.

[16] Niwińska A., Gałecki J. (2016). Current indications and methods of postoperative radiation therapy—Repetition before the exam. Oncol. Clin Pract..

[17] Censabella S., Claes S., Orlandini M., Braekers R., Thijs H., Bulens P. (2014). Retrospective study of radiotherapy-induced skin reactions in breast cancer patients: Reduced incidence of moist desquamation with a hydroactive colloid gel versus dexpanthenol. Eur. J. Oncol. Nurs..

[18] Sanchis A.G., González L.B., Carazo J.L.S., Partearroyo J.C.G., Martínez A.E., González A.V., Torrecilla J.L.L. (2017). Evaluation of acute skin toxicity in breast radiotherapy with a new quantitative approach. Radiother. Oncol..

[19] Rodenberg D.A., Chaet M.S., Bass R.C. (1995). Nitric Oxide: An Overview. Am. J. Surg..

[20] Nakamura Y., Yasuoka H. (2006). Nitric Oxide in Breast Cancer. Clin. Cancer Res..

[21] Anbar M. (1994). Hyperthermia of the cancerous breast: Analysis of mechanism. Cancer Lett..

[22] Amalric R., Giraud D., Altschuler C., Amalric F., Spitalier J.M., Brandone H., Ayme Y., Gardiol A.A. (1982). Does infrared thermography truly have a role in present day breast cancer management. Biomed. Thermol..

[23] Jones C.H., Parsons C.A. (1983). Thermography of the female breast. Diagnosis of Breast Disease.

[24] Head J.F., Lipari C.A., Elliot R.L. Comparison of mammography and breast infrared imaging: Sensitivity, specificity, false negatives, false positives, positive predictive value and negative predictive value. Proceedings of the First Joint BMES/EMBS Conference. 1999 IEEE Engineering in Medicine and Biology 21st Annual Conference and the 1999 Annual Fall Meeting of the Biomedical Engineering Society.

[25] Cholewka A., Kajewska J., Kawecki M., Sieroń-Stołtny K., Stanek A. (2017). How to use thermal imaging in venous insufficiency?. J. Therm. Anal. Calorimetry.

[26] Cholewka A., Drzazga Z., Sieroń A., Stanek A. (2010). Thermovision diagnostics in chosen spine diseases treated by whole body cryotherapy. J. Therm. Anal. Calorim..

[27] Cholewka A., Stanek A., Kwiatek S., Sieroń A., Drzazga Z. (2013). Does the temperature gradient correlate with the photodynamic diagnosis parameter numerical colour value (NCV)?. Photodiagn. Photodyn. Ther..

[28] Cholewka A., Stanek A., Klimas A., Sieroń A., Drzazga Z. (2013). Thermal imaging application in chronic venous disease: Pilot study. J. Anal. Calorim..

[29] Kajewska J., Cholewka A., Pająk J., Stanek A. The thermal imaging parameters in correlation with USG duplex parameters used in chronic venous disease of lower extremities diagnosis. Proceedings of the Quantitative InfraRed Thermography.

[30] Keyserlingk J.R., Ahlgren P.D. (1998). Infrared imaging of the breast; initial reappraisal using high—Resolution digital technology in 100 successive cases of stage 1 and 2 breast cancer. Breast J..

[31] Herman C., Cetingul M.P. (2011). Quantitative visualization and detection of skin cancer using dynamic thermal imaging. J. Vis. Exp..

[32] Head J.F., Wang F., Elliott R.L. (1993). Breast thermography is a noninvasive prognostic procedure that predicts tumor growth rate in breast cancer patients. Ann. N. Y. Acad. Sci..

[33] Ng E.Y.K., Sudharsan N.M. (2001). Numerical computation as a tool to aid thermographic Interpretation. J. Med. Eng. Technol..

[34] Kennedy D.A., Lee T., Seely D. (2009). A Comparative Review of Thermography as a Breast Cancer Screening Technique. Integr. Cancer Ther..

[35] Morales-Cervantes A., Kolosovas-Machuca E.S., Guevara E., Reducindo M.M., Hernández A.B.B., García M.R., González F.J. (2018). An automated method for the evaluation of breast cancer using infrared thermography. EXCLI J..

[36] Brzezińska D., Baic A., Cholewka A., Stankiewicz M., Ślosarek K., Bzowski J., Stanek A., Sieroń K. (2018). Zastosowanie obrazowania termicznego w diagnostyce nowotworów piersi. Inżynier I Fiz. Med..

[37] Plaza D., Baic A., Lange B., Stanek A., Szurko A., Sieroń K., Ślosarek K., Cholewka A. (2020). Zastosowanie obrazowania termicznego w ocenie efektów radioterapii u pacjentek po mastektomii. Inżynier I Fiz. Med..

[38] Plaza D., Baic A., Lange B., Stanek A., Ślosarek K., Kowalczyk A., Cholewka A. (2021). Correlation between Isotherms and Isodoses in Breast Cancer Radiotherapy—First Study. Int. J. Environ. Res. Public Health.

[39] Harper J.L., Franklin L.E., Jenrette J.M., Aguero E.G. (2004). Skin Toxicity During Breast Irradiation: Pathophysiology and Management. South. Med. J..

[40] Hymes S.R., Strom E.A., Fife C. (2006). Radiation dermatitis: Clinical presentation. pathophysiology and treatment 2006. J. Am. Acad Dermatol..

[41] Maillot O., Leduc N., Atallah V., Escarmant P., Petit A., Belhomme S., Sargos P., Vinh-Hung V. (2017). Evaluation of Acute Skin Toxicity of Breast Radiotherapy Using Thermography: Results of a Prospective Single-Centre Trial.

[42] Prasada S.S., Ramachandraa L., Kumarb V., Davea A., Mesthac L.K., Venkatarmani K. (2016). Evaluation of efficacy of thermographic breast imaging in breast cancer: A pilot study. Breast Dis..

[43] Lilla C., Ambrosone C.B., Kropp S., Helmbold I., Schmezer P., Von Fournier D., Haase W., Sautter-Bihl M.-L., Wenz F., Chang-Claude J. (2007). Predictive factors for late normal tissue complications following radiotherapy for breast cancer. Breast Cancer Res. Treat..

[44] Chuang H.-Y., Hou M.-F., Luo K.-H., Wei S.-Y., Huang M.-Y., Su S.-J., Kuo H.-Y., Yuan S.-S., Chen G.-S., Hu S.C.-S. (2015). RTOG, CTCAE and WHO criteria for acute radiation dermatitis correlate with cutaneous blood flow measurements. Breast.

[45] Zhu W., Jia L., Chen G., Li X., Meng X., Xing L., Zhao H. (2019). Relationships between the changes of skin temperature and radiation skin injury. Int. J. Hyperth..

[46] Greco F., Quarta L.G., Grasso R.F., Beomonte Zobel B., Mallio C.A. (2020). Increased visceral adipose tissue in clear cell renal cell carcinoma with and without peritumoral collateral vessels. Br. J. Radiol..

[47] Vavassis P., Gelinas M., Chabot Tr J., Nguyen-Tân P.F. (2008). Phase 2 study of silver leaf dressing for treatment of radiation-induced dermatitis in patients receiving radiotherapy to the head and neck. J. Otolaryngol. Head Neck Surg..

[48] Omidvari S., Saboori H., Mohammadianpanah M., Mosalaei A., Ahmadloo N., Mosleh-Shirazi M.A., Jowkar F., Namaz S. (2007). Topical betamethasone for prevention of radiation dermatitis. Indian J. Dermatol. Venereol. Leprol..

[49] Trotti A., Bentzen S.M. (2004). The need for adverse effects reporting standards in oncology clinical trials. J. Clin. Oncol..

[50] Chan R.J., Larsen E., Chan P. (2012). Re-examining the Evidence in Radiation Dermatitis Management Literature: An Overview and a Critical Appraisal of Systematic Reviews. Int. J. Radiat. Oncol..

